# *Myroides* species infection in a chronic venous stasis ulcer: A rare multidrug-resistant opportunistic pathogen

**DOI:** 10.1016/j.idcr.2026.e02581

**Published:** 2026-04-20

**Authors:** Patrick Hunt, Kimberly Huynh, Matthew Papiernik, Ashley Parker, Sofia Barajas, Sully Paika, Randy Odero

**Affiliations:** a Midwestern University Arizona College of Osteopathic Medicine, Glendale, AZ, USA; b Department of Internal Medicine, Abrazo Arizona Heart Hospital, Phoenix, AZ, USA; c Department of Infectious Disease, Abrazo Arizona Heart Hospital, Phoenix, AZ, USA

**Keywords:** *Myroides odoratimimus*, Venous stasis ulcer, Multidrug resistance, Chronic wound infection, Opportunistic infection, Cellulitis

## Abstract

*Myroides* species, previously known as *Flavobacterium*, are rare, opportunistic Gram-negative bacilli primarily found in environmental sources such as water and soil and are infrequently implicated in human infection. When present, infection is often associated with multidrug- resistance and immunocompromised hosts. Cutaneous wound infections caused by *Myroides* spp. remain poorly described in the literature. We report a case of *Myroides odoratus/odoratimimus* isolated from chronic bilateral venous stasis ulcers in a 78-year-old man with significant cardiovascular disease and chronic venous insufficiency who presented with acute bilateral lower-extremity cellulitis and sepsis. The patient exhibited hypotension, tachycardia, acute kidney injury, and malodorous wound drainage, prompting initiation of broad-spectrum intravenous antimicrobial therapy. Wound cultures subsequently identified *Myroides* spp. with concern for multidrug-resistance, and the patient demonstrated clinical improvement with continued culture-directed management. This case emphasizes the ability of *Myroides* spp. to cause acute severe infection in chronic venous ulcers, highlighting the importance of early recognition and microbiologic diagnosis in guiding appropriate therapy.

## Introduction

*Myroides* species are aerobic, yellow-pigmented, non-motile, non-fermenting Gram-negative bacilli formerly classified as *Flavobacterium odoratum* and are widely distributed in environmental sources such as soil and water [1], [2]. Historically regarded as low-virulence opportunistic organisms, *Myroides* spp. have increasingly been recognized as clinically significant pathogens responsible for a broad spectrum of infections, including skin and soft tissue infections, urinary tract infections, bacteremia, endocarditis, and pneumonia [1], [2]. The rising number of reported cases likely reflects both improved microbiologic identification techniques, particularly MALDI-TOF mass spectrometry and molecular sequencing, and an expanding population of susceptible hosts [1], [3].

Risk factors for *Myroides* infection include prolonged hospitalization, intensive care unit exposure, invasive procedures, prior broad-spectrum antibiotic therapy, and underlying comorbidities such as diabetes mellitus, cerebrovascular disease, liver disease, and immunosuppression [4], [5]. Although cutaneous manifestations have most commonly been reported in severely immunocompromised patients, an increasing number of cases demonstrate that *Myroides* spp. can also cause significant soft tissue infections in immunocompetent individuals, ranging from uncomplicated cellulitis to fulminant necrotizing fasciitis [2], [5]. Given their resistance profile, potential for severe infection, and increasing recognition in clinical practice, early identification of *Myroides* species and susceptibility-guided antimicrobial therapy are essential. Awareness of this uncommon pathogen is particularly important when evaluating acute skin and soft tissue infections involving chronic wounds or atypical clinical presentations.

## Case

**Fig. 1:**
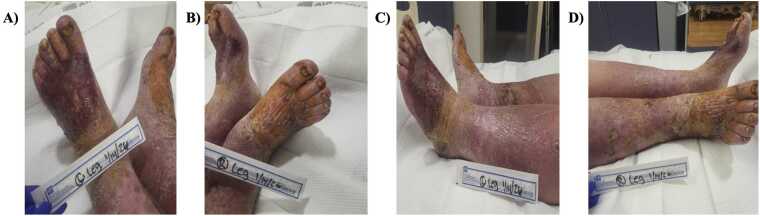
Appearance of venous stasis cellulitis on hospital day 1. **(A)** Left lower extremity wounds photographed from a superior view on January 14, 2026 (hospital day 1). **(B)** Right lower extremity wounds photographed from a superior view on January 14, 2026 (hospital day 1). **(C)** Left lower extremity wounds photographed from a lateral view on January 14, 2026 (hospital day 1). **(D)** Right lower extremity wounds photographed from a lateral view on January 14, 2026 (hospital day 1).

A 78-year-old man with chronic venous insufficiency complicated by bilateral venous stasis ulcers, peripheral arterial disease status-post left anterior tibial artery PCI, atrial fibrillation on anticoagulation, heart failure with reduced ejection fraction (25%), severe aortic stenosis, mild-moderate aortic and mitral regurgitation, emphysema, and remote tobacco use presented with progressively worsening bilateral lower-extremity and foot ulcers associated with increasing drainage, skin breakdown, erythema, pain, and functional limitation after inability to obtain outpatient wound care. On arrival, he appeared globally ill and fatigued. Initial evaluation demonstrated concern for bilateral lower-extremity cellulitis in the setting of chronic venous and ischemic ulcerations (Fig. 1). Laboratory studies showed normal leukocyte count, sodium 127 mmol/L, creatinine 1.50 mg/dL, blood urea nitrogen 26 mg/dL, and lactic acid 1.8 mmol/L. Blood and wound cultures were obtained, and empiric intravenous vancomycin and piperacillin-tazobactam were initiated.

**Fig. 2:**
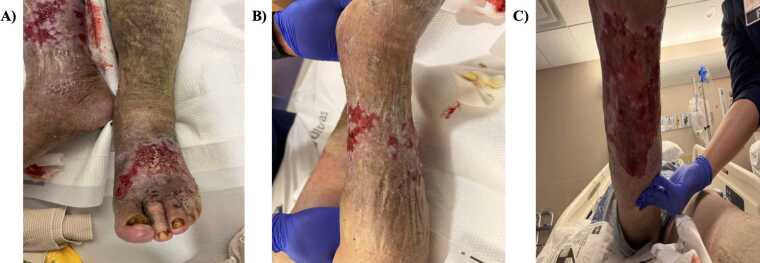
Appearance of wounds on hospital day 9, one day prior to discharge. **(A)** Left lower extremity wounds photographed from a superior view on January 25, 2026 (hospital day 9). **(B)** Right lower extremity wounds photographed from a superior view on January 25, 2026 (hospital day 9). **(C)** Right lower extremity wound photographed from an inferior view on January 25, 2026 (hospital day 9).

Shortly after admission, the patient was noted to be hypotensive (85/70 mmHg) and tachycardic in atrial fibrillation without rapid ventricular response, with intermittent metabolic encephalopathy, raising concern for sepsis. He received a 1-l normal saline bolus followed by cautious maintenance fluids at 75 mL/h due to underlying cardiac disease, and the sepsis bundle was initiated. Physical examination revealed extensive bilateral lower-extremity edema with superficial venous ulcers producing foul-smelling greenish drainage, maceration, increased warmth, and copious exudate without crepitus or necrosis (Fig. 1). Over the subsequent hospital course, the patient remained afebrile and clinically improved on intravenous antibiotics, with improving renal function and wound appearance (Fig. 2). Wound cultures demonstrated predominant and heavy growth of *Myroides odoratus/odoratimimus* admist mixed flora, and antimicrobial susceptibilities were requested due to concern for multidrug-resistance (Table 1).

**Table 1:** Antimicrobial susceptibility profile o*f Myroides odoratus/odoratimimus* isolated from wound culture.

**Wound Culture**	***Myroides odoratus/odoratimimus*****Susceptibility**	
**Antimicrobial Agent**	**Interpretation**	**MIC (µg/mL)**
Amikacin	S	≤ 16
Aztreonam	S	8
Cefepime	S	4
Cefotaxime	I	16
Ceftazidime	S	4
Ceftriaxone	I	32
Ciprofloxacin	R	> 2
Gentamicin	S	≤ 2
Levofloxacin	S	1
Meropenem	S	≤ 1
Piperacillin/Tazobactam	S	≤ 8
Tetracycline	R	> 8
Tobramycin	S	≤ 2
Trimethoprim/Sulfamethoxazole	R	> 2/38

**Abbreviations:** MIC, minimum inhibitory concentration; S, susceptible; I, intermediate; R, resistant.

Susceptibility testing revealed a multidrug-resistant profile, with resistance to ciprofloxacin, trimethoprim-sulfamethoxazole, and tetracycline, qualifying as MDR with non-susceptibility to at least one agent in three or more antimicrobial categ while retaining susceptibility to piperacillin-tazobactam, cefepime, meropenem, and aminoglycosides (Table 1). Given the absence of gram-positive organisms requiring continued glycopeptide coverage and the organism’s susceptibility profile, vancomycin was discontinued, and antimicrobial therapy was narrowed to piperacillin-tazobactam monotherapy. The patient continued to demonstrate clinical improvement following de-escalation, with further reduction in wound drainage, erythema, and edema, as well as stabilization of renal function and electrolytes. His favorable response supported the continued use of piperacillin-tazobactam until transition to outpatient management.

Over the remainder of his hospitalization, the patient continued to demonstrate clinical improvement with intravenous piperacillin-tazobactam and ongoing wound care, without evidence of bacteremia or recurrent sepsis. His hospital course was complicated by persistent atrial fibrillation, progressive systolic dysfunction, and severe aortic stenosis, prompting coronary angiography with percutaneous coronary intervention to the left anterior descending and right coronary arteries, as well as transesophageal echocardiography-guided direct current cardioversion, after which he remained in sinus rhythm. Rising liver enzymes were noted later in the course and were attributed to medication-related hepatotoxicity, with interval improvement following discontinuation of piperacillin-tazobactam. After a ten-day hospital course with clinical resolution and high likelihood of colonization in chronic venous stasis ulcers, antibiotics were discontinued at discharge in favor of continued wound care and close follow-up. The patient was deemed medically stable for discharge to a skilled nursing facility for continued wound care, rehabilitation, and laboratory monitoring, with plans for outpatient evaluation for transcatheter aortic valve replacement.

## Discussion

Over the past two decades, *Myroides* spp. have emerged as clinically relevant opportunistic pathogens, in part due to their resistance to multiple antimicrobial classes, including ꞵ-lactams and aminoglycosides, complicating inpatient management [6], [7]. In a review by Khan et al. in 2023, *Myroides* spp. have been implicated in 97 reported cases in the literature, and only 18 cases reported cutaneous infections [7]. To our knowledge, this report represents the 19th documented case of *Myroides*-associated cutaneous infection, further expanding on the limited clinical experience with this uncommon pathogen. Most published cases of cutaneous and soft tissue infections caused by *Myroides* spp. were found primarily in immunocompromised patients or in association with unsanitary environmental or animal exposure [7], [8], [9], [10], [11]. Chronic wounds, particularly venous stasis ulcers, provide a plausible portal of entry for infection due to tissue hypoxia, impaired local immune defenses, and ongoing exposure to environmental organisms [9], [10], [11], [12], [13]. However, *Myroides* spp. have only rarely been implicated in this setting. Increased recognition in recent years is likely attributable to advances in microbial identification, particularly the widespread adoption of matrix-assisted laser desorption/ionization time-of-flight (MALDI-TOF) mass spectrometry [6], [7]. This case is noteworthy in that it demonstrates sepsis in a patient without overt immunosuppression or exposure to known sources of infection. Upon further investigation of a source of *Myroides* infection, the patient denied any pets at home or recent significant outdoor activities, including gardening or sewage water exposure, as was previously reported to be the mechanism of inoculation [7], [8], [9], [10], [11]. In the absence of overt sources of exposure, the patient’s chronic wound care may have contributed through the introduction of water-associated organisms during wound cleansing, dressing changes, or debridement. These findings suggest that chronic wound care practices may represent a potential route for *Myroides* spp. inoculation, warranting a heightened clinical suspicion for this pathogen in patients with refractory venous stasis ulcers.

The patient’s presentation highlights the pathogenic potential of *Myroides* spp. in hosts with significant comorbidity burden in the absence of classic immunosuppressive conditions. Although advanced age, cardiovascular disease, and chronic venous insufficiency contributed to impaired immunity, the patient did not present with malignancy, neutropenia, organ transplantation, or chronic immunosuppressive therapy, factors described in previous reports. Recent reports by Juusola et al. and Do et al. depict similar infections in immunocompetent patients with chronic venous ulcers, suggesting that *Myroides* spp. infections may not be exclusively limited to immunosuppressed patients and may be underrecognized in those with chronic wounds or vascular disease [7], [8], [9], [10], [11]. The development of hypotension, metabolic encephalopathy, and acute kidney injury in this case further emphasizes the need for early recognition and aggressive management.

A defining challenge in the management of *Myroides* infections is their intrinsic multidrug-resistance. These organisms harbor chromosomally encoded metallo-β-lactamases, including MUS-1 in *M. odoratimimus* and TUS-1 in *M. odoratus*, which confer resistance to many β-lactam antibiotics, aminoglycosides, and carbapenems [1], [12], [13]. Susceptibility studies consistently demonstrate high resistance rates to cephalosporins, aminoglycosides, and carbapenems, with *M. odoratus* generally exhibiting greater resistance than *M. odoratimimus* [1], [4], [12]. Fluoroquinolones, particularly levofloxacin, and trimethoprim-sulfamethoxazole have shown comparatively greater in vitro activity [4], [12]. Interestingly, the isolate in this case demonstrated susceptibility to piperacillin-tazobactam, cefepime, meropenem, and aminoglycosides, with resistance to ciprofloxacin, tetracycline, and trimethoprim-sulfamethoxazole. Consistent with Do et al., our isolate was sussceptible to piperacillin-tazobactam, but exhibited atypical resistance to ciprofloxacin and trimethoprim-sulfamethoxazole [11]. This highlights the heterogeneity of antimicrobial resistance among *Myroides* spp. and reinforces the necessity of organism-specific susceptibility testing rather than reliance on historical resistance patterns alone. While fluoroquinolones and trimethoprim-sulfamethoxazole are often cited as preferred agents when susceptible, these medications carry significant adverse side effects, particularly in patients with significant comorbidities who are at risk for medication-related toxicity, as evidenced by the transient hepatotoxicity observed in this patient’s course [4], [13]. The patient’s favorable clinical response following de-escalation to piperacillin-tazobactam monotherapy further supports the role of culture-directed therapy in managing *Myroides* infections.

This case highlights that *Myroides* spp., though rare, should be considered potential pathogens in patients presenting with infected chronic venous stasis ulcers, even in the absence of profound immunosuppression or classical environmental exposures. Given the organism’s variable and often multidrug-resistant susceptibility profile, early wound culture and susceptibility testing are essential to guide appropriate antimicrobial therapy. Heightened awareness and consideration of *Myroides* spp., particularly in the setting of chronic wounds, may facilitate timely diagnosis, targeted treatment, and improved clinical outcomes.

## CRediT authorship contribution statement

**Ashley Parker:** Writing – review & editing. **Sully Paika:** Writing – review & editing. **Randy Odero:** Supervision. **Sofia Barjas:** Writing – review & editing. **Patrick Hunt:** Conceptualization, Data curation, Writing – original draft, Writing – review & editing. **Kimberly Huynh:** Writing – original draft, Writing – review & editing. **Matthew Papiernik:** Writing – review & editing.

## Author agreement

The undersigned authors certify that:

1. All authors have read and approved the final version of the manuscript being submitted.
2. The manuscript is original work and has not been previously published.
3. The manuscript is not under consideration for publication elsewhere.
4. All authors agree to its submission to IDCases.

## Consent

Written informed consent was obtained from the patient for publication of this case report and accompanying images.

## Funding

No funding was received for this work.

## Conflict of Interest Statement

The authors declare no conflicts of interest.

## Declaration of Competing Interest

The authors declare that they have no known competing financial interests or personal relationships that could have appeared to influence the work reported in this paper.

## References

[1] Colín-Castro C.A., Ortiz-Álvarez J.M., Hernández-Pérez C.F. (2024). Myroides species, pathogenic spectrum and clinical microbiology site in Mexican isolates. PLoS One.

[2] Benedetti P., Rassu M., Pavan G., Sefton A., Pellizzer G. (2011). Septic shock, pneumonia, and soft tissue infection due to Myroides odoratimimus: report of a case and review of Myroides infections. Infection.

[3] Schröttner P., Rudolph W.W., Eing B.R., Bertram S., Gunzer F. (2014). Comparison of VITEK2, MALDI-TOF MS, and 16S rDNA sequencing for identification of Myroides odoratus and Myroides odoratimimus. Diagn Microbiol Infect Dis.

[4] Gülmez A., Ceylan A.N., Özalp O. (2023). An increasing threat in intensive care units: evaluation of multidrug-resistant Myroides spp. infections and risk factors. J Hosp Infect.

[5] Crum-Cianflone N.F., Matson R.W., Ballon-Landa G. (2014). Fatal case of necrotizing fasciitis due to Myroides odoratus. Infection.

[6] Gunzer F., Rudolph W.W., Bunk B. (2018). Whole-genome sequencing of a large collection of Myroides odoratimimus and Myroides odoratus isolates and antimicrobial susceptibility studies. Emerg Microbes Infect.

[7] Khan U., Pandey E., Gandham N., Das N., Mukhida S., Kannuri S. (2023). A case series and literature review of infections due to Myroides spp.: identification of contributing factors and emerging antibiotic susceptibility trends. Access Microbiol.

[8] Juusola T., Rahkonen M., Aho-Laukkanen E., Mäki-Koivisto V., Junttila I.S. (2025). Poor effect of empiric antibiotic treatment of Gram-negative bacteria on Myroides spp.: a case report and literature review. Case Rep Infect Dis.

[9] Maraki S., Sarchianaki E., Barbagadakis S. (2012). Myroides odoratimimus soft tissue infection in an immunocompetent child following a pig bite: case report and literature review. Braz J Infect Dis.

[10] Foo R.M., Nanavati S.M., Samuel A., Lamm R., Upadhyay S. (2020). Gardener's nightmare: a rare case of Myroides-induced septic shock. Cureus.

[11] Do S., Rebentish A., Ravichandran Kumar P. (2023). Case report of Myroides odoratimimus cellulitis in chronic venous stasis dermatitis with literature review. Cureus.

[12] Oyardi O., Eltimur T., Demir E.S. (2023). Antibacterial and antibiofilm activities of ceragenins alone and in combination with levofloxacin against multidrug-resistant Myroides spp. clinical isolates from patients with urinary tract infections. Curr Microbiol.

[13] Xu S., Chen Y., Fu Z. (2018). New subclass B1 metallo-β-lactamase gene from a clinical pathogenic Myroides odoratus strain. Micro Drug Resist.

